# Exploring the Effects of Greek Yogurt Supplementation and Exercise Training on Serum Lithium and Its Relationship With Musculoskeletal Outcomes in Men

**DOI:** 10.3389/fnut.2021.798036

**Published:** 2021-12-22

**Authors:** Ryan W. Baranowski, Lauren E. Skelly, Andrea R. Josse, Val A. Fajardo

**Affiliations:** ^1^Centre for Bone and Muscle Health, Brock University, St. Catharines, ON, Canada; ^2^Department of Kinesiology, Faculty of Applied Health Sciences, Brock University, St. Catharines, ON, Canada; ^3^School of Kinesiology and Health Science, Faculty of Health, York University, Toronto, ON, Canada

**Keywords:** body composition, strength, bone turnover, dairy, resistance training, P1NP, CTX

## Abstract

Dairy products can act as a dietary source of lithium (Li), and a recent study in university-aged males demonstrated that Greek yogurt (GY) supplementation augmented gains in fat free mass, strength and bone formation after 12 weeks of resistance exercise training compared to carbohydrate (CHO) pudding supplementation. Here, we performed secondary analyses to explore whether GY would alter serum Li levels and whether changes in serum Li would associate with changes in body composition, strength, and bone turnover markers. Results show that the GY group maintained serum Li levels after exercise training, whereas the CHO group did not. Maintaining/elevating serum Li levels was also associated with greater gains in strength and reductions in bone resorption. However, controlling for other dietary factors in GY such as protein and calcium weakened these associations. Thus, future studies should assess the causative role, if any, of dietary Li alone on strength and bone resorption in humans.

## Introduction

Lithium (Li) is a monovalent cation commonly prescribed for bipolar disorder; however, psychiatric use of Li requires a dose high enough (~0.8 mM serum Li) to penetrate the blood brain barrier and to exert benefits on mental health (1). Unfortunately, these high doses of Li have been associated with adverse effects to peripheral organs such as the kidney, thyroid, and gastrointestinal tract (2–5). Conversely, studies in rodent models have shown that low dose Li supplementation—with levels well below those used for bipolar disorder–can provide several physiological benefits. For example, in 2010, Choi and colleagues showed that 10 mg/kg/day supplementation of Li, which we have shown results in a serum [Li] of 0.02 mM (6), attenuated high-fat diet induced obesity and atherosclerosis in mice (7). With this same dose, we have shown that 6-weeks of Li supplementation can enhance skeletal muscle force production and osteogenic signaling that favors bone formation over resorption (6, 8). In all, it is possible that the adverse effects associated with chronic use of high dose Li therapy may have led to an underappreciation of the benefits of low dose Li supplementation; however, whether these benefits translate to humans remains unknown.

Aside from therapy, Li can be naturally obtained from the diet with main food sources including cereals, potatoes, tomatoes, cabbage, dairy products (i.e., milk and yogurt), and in some mineral- and tap-water (depending on geographical location) (9–12). In a recent study by Bridge et al. (13), Greek yogurt (GY) supplementation enhanced fat free mass (FFM) gains, biceps thickness, and total strength after a 12-week resistance and plyometric exercise program in untrained, university-aged males (13). In addition, another analysis from this study discovered that GY supplementation increased bone formation demonstrated by an increased concentration of the bone formation marker procollagen type 1 N-terminal propeptide (P1NP) and an increase in the ratio of P1NP to the bone resorption marker β-isomerized carboxy-terminal cross-linking telopeptides (CTX) in serum after the 12-week exercise intervention (14). Given that Li can be found in dairy products such as yogurt (0.07 ± 0.04 mg/kg) (9), in this exploratory analysis we examined whether GY supplementation altered serum Li levels, and whether changes in serum Li were associated with various changes in body composition, strength, and markers of bone turnover after 12 weeks of exercise training in young males.

## Materials and Methods

### Participants, Ethics Approval, and Study Design

Thirty healthy (free of medical conditions), young, untrained, adult (18–25 y) males were recruited from Brock University (Ontario, Canada) between July 2017 and August 2018 to participate in the *Brock Exercise And Supplement Trial* (BEAST) as previously described (13). All subjects were informed of potential study risks, and written informed consent was obtained. The original BEAST Study was approved by the Brock University Biosciences Research Ethics Board (REB#16-295) and registered at clinicaltrials.gov (NCT03196856), and these exploratory analyses were subsequently approved (REB#20-172).

This parallel, randomized, controlled, intervention study divided subjects into two groups: a Greek yogurt group (GY; *n* = 15) and a Placebo carbohydrate (CHO) pudding group (*n* = 15). The GY group consumed 200 g of Oikos 0% fat, plain GY (110 Kcals, 20 g protein, 8 g CHO; Danone Canada Inc., Boucherville, Quebec) 3 times/day on training days (immediately post-exercise, 1-h post-exercise, and before bed) and 150 g 2 times/day on non-training days (breakfast and before bed). The CHO group consumed 47 g of an isoenergetic, chocolate flavored, CHO-based semi-solid pudding (110 Kcals, 0 g protein, 28 g CHO) that was designed to resemble the consistency and texture of GY on the same schedule as the GY group. The CHO supplement's contents were kept discreet to participants. Many participants believed this supplement was the “test product” and that it may have contained muscle-supporting nutrients like protein. The 12-week exercise protocol consisted of supervised exercise training sessions 3 days/week. Sessions included full-body resistance training at 70% of 1-repetition maximum strength (2/d week) and plyometric training (1/d week). All participants were provided with the same information and advice to help them compensate for the added calories consumed from the supplements, as a means to avoid participants being in a positive energy balance. Additional details regarding the exercise training and supplementation program have been published elsewhere (13).

### Body Composition, Biceps Thickness, Strength, Markers of Bone Turnover, and Dietary Records

Pre- and post-study measures of body composition (body mass, FFM, fat mass and % body fat *via* Bodpod [Cosmed USA Inc. Chicago, Il]), biceps and quadriceps muscle thickness (*via* ultrasonography [GE Medical Systems, Ultrasound Vivid I portable, Milwaukee, WI, USA]), total strength (combined 1-repetition maximum scores of chest press, seated row, leg extension, and hamstring curl) and circulating levels of the bone turnover biomarkers P1NP (Roche Diagnostics; cat. no. 03141071 190 *via* Roche Elecsys e411 automated analyzer) and CTX (β-CrossLaps; cat. no.: 11972308122 *via* Roche Cobas e602 automated analyzer) were assessed as previously described (13, 14). Dietary calcium, protein, phosphorus and potassium intake were all measured using 7-day (at baseline) and 3-day (during the 12th week of training) food diaries (13).

### Serum Lithium Analysis

Serum and GY Li concentrations were measured using inductively coupled plasma mass spectrometry (ICP-MS) as previously described (6).

### Statistics

Baseline comparisons between CHO and GY were assessed using an independent *t*-test. Assumptions of normality for all data were tested using a D'Agostino & Pearson test. A two-way repeated measures ANOVA, with a Bonferroni *post-hoc* test was used to examine changes in serum Li pre- and post-exercise training in the GY and CHO groups. Pearson's or Spearman's correlational analyses were done, depending on tests for normality, to examine the association between absolute changes in serum Li and changes in body mass, body composition, muscle thickness, strength, and markers of bone turnover (change = post-pre) using pooled (GY + CHO) data. Partial correlations were performed to control for change in components of dairy, including calcium, protein, phosphorus, and potassium. Analyses were only conducted in 25 of the total 30 participants as 2 did not have post-exercise blood samples (GY *n* = 1; CHO *n* = 1), 2 did not complete the full 12 weeks of training (GY *n* = 1; CHO *n* = 1), and one did not provide a 3-day post-intervention food diary (GY *n* = 1). Thus, from the original allocation of *n* = 15 per group, we analyzed datasets from 12 GY and 13 CHO participants. Statistical outliers for each dependent variable detected through a ROUT method (*Q* = 2%) were removed (0–2 samples) prior to correlational analyses. SPSS statistical software (IBM) was used for all statistical analyses and significance was set at *p* ≤ 0.05.

## Results

**Figure 1:**
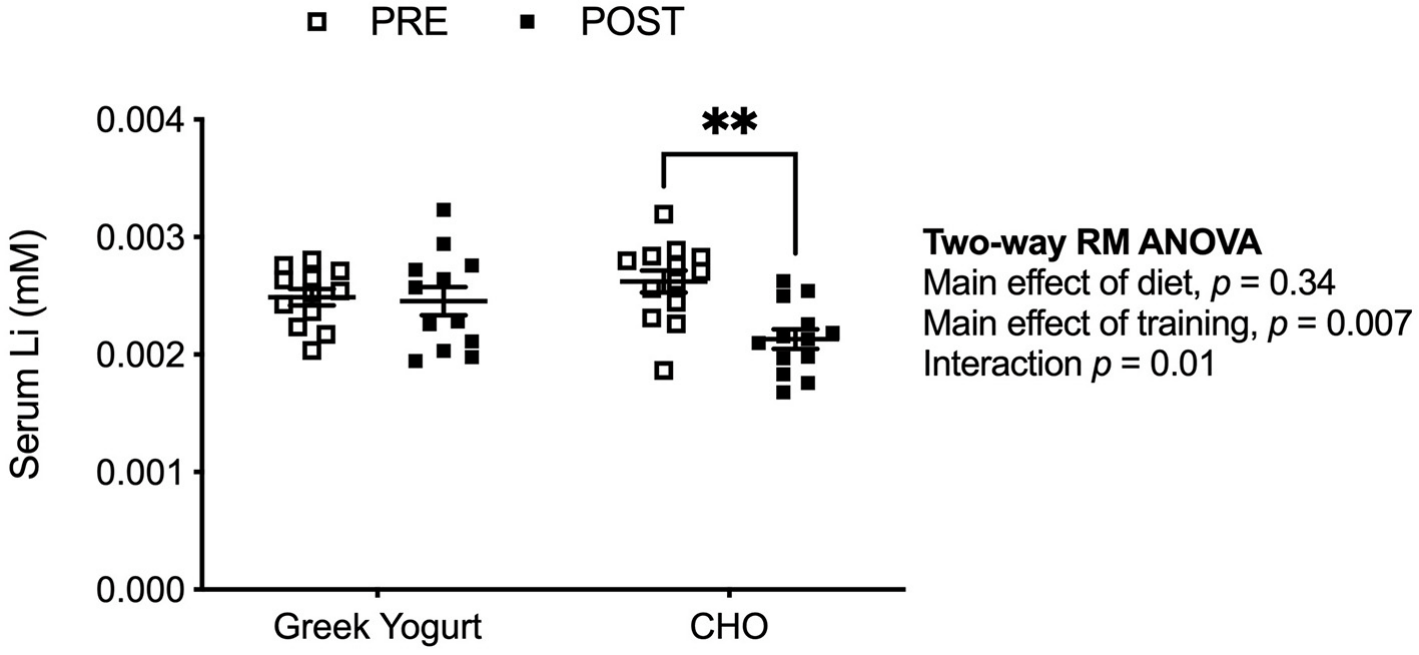
Serum Li concentrations are lowered after exercise training but only in the CHO group and not the GY group. **p < 0.01 using a two-way repeated measures ANOVA with a Bonferroni *post-hoc* test (GY, *n* = 12; CHO, *n* = 13 per group).

Li content in Greek yogurt was measured and found to be 0.02 mg/kg (0.0025 mM) (9). In Figure 1, we examined serum Li concentration pre- and post-exercise training in the GY and CHO groups. Two-way repeated measures ANOVA revealed a significant interaction between diet and training, which indicated that the CHO group displayed a reduction in serum Li after training, whereas the GY group did not (Figure 1).

There were no differences between groups in baseline musculoskeletal variables or dietary intakes of protein, calcium, potassium and phosphorus (Table 1). Changes in serum Li levels did not correlate with changes in body mass, body composition or muscle size, but a significant positive correlation was observed with changes in total strength (Table 2). While there was no association between changes in serum Li and changes in P1NP, a significant and negative association between changes in serum Li and CTX was observed. This led to a significant positive association between changes in serum Li and the P1NP:CTX ratio. Partial correlational analyses revealed that even after controlling for changes in other dietary dairy components, protein, calcium, phosphorus and potassium, significant associations between changes in serum Li and total strength and CTX were maintained (Table 3). However, the relationship between changes in serum Li and the P1NP:CTX ratio was no longer statistically significant.

**Table 1:** Baseline characteristics of study participants.

	**CHO (*n* = 13)**	**GY (*n* = 12)**	***p***
Body mass (kg)	69.4 ± 3.1	69.8 ± 3.0	0.93
Fat-free mass (kg)	57.6 ± 2.1	60.4 ± 2.4	0.39
Fat mass (kg)	11.8 ± 1.7	9.3 ± 1.1	0.25
Body fat (%)	16.2 ± 2.0	13.1 ± 1.2	0.21
Biceps thickness (cm)	2.8 ± 0.1	2.8 ± 0.1	0.96
Quadriceps thickness (cm)	3.6 ± 0.2	3.8 ± 0.2	0.50
Total strength (kg)	383.8 ± 18.7	364.9 ± 24.5	0.54
P1NP (μg/L)	115.1 ± 12.6	101.0 ± 9.7	0.39
CTX (ng/L)	856.8 ± 78.9	815.0 ± 65.1	0.69
P1NP:CTX	0.14 ± 0.01	0.13 ± 0.01	0.60
Dietary calcium intake (mg)	676 ± 65	698 ± 75	0.82
Dietary protein intake (g/kg)	1.28 ± 0.07	1.30 ± 0.09	0.74
Dietary phosphorus intake (mg)	667 ± 99	744 ± 81	0.56
Dietary potassium intake (mg)	1573 ± 198	1529 ± 189	0.87

*Values are presented as mean ± SEM. CTX, β-isomerized carboxy-terminal cross-linking telopeptides; P1NP, procollagen type 1 N-terminal propeptide. The p-value was achieved using a Student's unpaired t-test*.

**Table 2:** Correlational analyses with changes in serum Li (mM) and changes in musculoskeletal variables.

	***r***	***p***	***n***
Δbody mass (kg)	0.11	0.59	25
Δfat-free mass (kg)	0.26	0.20	25
Δfat mass (kg)	0.05	0.80	24
Δbody fat (%)	−0.07	0.75	25
Δbiceps thickness (cm)	0.34	0.11	23
Δquadriceps thickness (cm)	−0.02	0.91	24
Δtotal strength (kg)	0.57	0.004	23
ΔP1NP (μg/L)	0.20	0.34	25
ΔCTX (ng/L)	−0.51	0.009	25
ΔP1NP:CTX	0.52	0.008	24

*CTX, β-isomerized carboxy-terminal cross-linking telopeptides; P1NP, procollagen type 1 N-terminal propeptide*.

**Table 3:** Partial correlational analyses with changes in serum Li (mM) and changes in total strength and bone turnover markers while controlling for dietary protein, calcium, phosphorus, and potassium intakes.

	***r***	***p***	***n***
Δtotal strength (kg)	0.48	0.04	23
ΔCTX (ng/L)	−0.45	0.04	25
ΔP1NP:CTX	0.27	0.26	24

*CTX, β-isomerized carboxy-terminal cross-linking telopeptides; P1NP, procollagen type 1 N-terminal propeptide*.

## Discussion

In this pilot/exploratory study, we questioned whether GY supplementation would alter serum Li levels after 12 weeks of exercise training, and whether changes in serum Li would correlate with changes in body composition, muscle thickness, total strength and markers of bone turnover in young males. We first demonstrated that serum Li levels were lower with exercise training, and while we do not have an exact explanation for this observation, we suspect that Li secretion through sweat (i.e., with repeated exercise) could play a role (15). Importantly, the reduction in serum Li was only observed in the CHO group and not the group supplemented with GY. As dairy products such as yogurt can act as a source of dietary Li (9), this result is not entirely surprising. In fact, post serum Li concentration in the GY group was closely matched with our measured concentration of Li in the actual GY provided to the subjects (0.0025 mM), which is within the range previously reported for dairy products (9). Though we did not measure Li in the CHO pudding, we suspect that, given its formulation and composition (2 parts maltodextrin, 1 part chocolate pudding powder and water), levels of Li would be negligible and possibly even below the level of detection. It is also possible that the CHO pudding itself may have a serum Li lowering effect or that its consumption replaced other Li-containing foods in the diet; however, this requires further investigation.

In the recent results from the BEAST study intervention, GY supplementation was shown to augment the gains in total strength and FFM and improve bone formation with exercise training (13, 14). Though our results showed that changes in serum Li levels did not associate with changes in body composition (i.e., FFM or body fat%) or muscle size, we did find significant associations between changes in serum Li with changes in total strength and markers of bone turnover. Changes in serum Li positively correlated with total strength even after controlling for other nutrients found in dairy products such as protein, calcium, phosphorus, and potassium. This suggests that those that maintained serum Li exhibited greater strength gains with exercise despite no influence of serum Li on muscle size or FFM. Nonetheless, this result is consistent with our previous work in rodents, where low dose Li supplementation increased specific force production in the murine soleus and extensor digitorum longus without any apparent changes in muscle mass (8). With respect to bone turnover, our findings indicate that changes in serum Li may be related to bone resorption, as it was negatively associated with changes in CTX even after controlling for other dietary dairy components. This result is also consistent with our previous rodent work showing that low dose Li supplementation increased the osteoprotegerin:RANKL ratio in the murine femur, which in theory would lead to less osteoclast differentiation/activity (6). When expressed as a ratio, P1NP:CTX provides a biomarker of bone collagen turnover (formation over resorption); and because of Li's negative association with CTX, we also found a significant positive association between changes in serum Li and changes in the P1NP:CTX ratio. However, after controlling for other dietary dairy components such as protein, calcium, phosphorus and potassium, this relationship was no longer significant. This was likely due to other dairy components, namely protein (*r* = 0.50, *p* = 0.01) and calcium (*r* = 0.45, *p* = 0.03), that were both positively correlated with P1NP:CTX, accounting for some of the independent association with serum Li. This was to be expected as other factors in dairy undoubtedly influence bone turnover (16, 17). Of particular importance is dietary protein as it can stimulate insulin-like growth factor 1 expression, which itself enhances bone mineralization and osteoblast differentiation and activation (18–20). In all, while we acknowledge that there are other factors in dairy that contribute to musculoskeletal strength and structure, our findings suggest that dietary Li within dairy may be an additional component worth further exploring.

The overall purpose of this pilot study was to form the basis of further work that will examine the role of dietary Li on musculoskeletal structure and function with and without exercise training. There were some limitations to our present study that should be addressed in the future. First, we could only speculate that the reduction in serum Li concentration observed after exercise training in the CHO group occurred through excessive Li secretion (i.e., sweat). Future studies should specifically measure the amount of Li lost in sweat and urinary secretions. Second, we did not measure the Li concentration in the CHO pudding, however due to the formulation of it, we expect that the Li levels would be negligible. Thirdly, participants recorded their food intake for a fewer number of days (3 days) during the 12th week of the intervention compared with baseline (7 days). We also did not control for Li in the dietary record. This is due to limitations in current software used to assess diet records that do not include the capacity to accurately measure Li intake. Nevertheless, a strength of our study was that we measured serum Li concentrations pre- and post-training. The use of muscle biopsies in this study could have provided additional mechanistic insight on the apparent associations between strength gains and serum Li through the examination of fiber type composition, cross-sectional area, single-fiber contractility, and various biochemical signaling pathways. For example, in rodents we have shown that low dose Li supplementation augments specific force production by inhibiting an enzyme called glycogen synthase kinase 3 (GSK3) (8), and therefore, future studies could also examine GSK3 activation in muscle biopsies with dietary Li supplementation. Though the underlying mechanism behind the apparent relationship between strength and Li in rodents and humans remains unknown, *in vitro*, we have shown that Li enhances myoblast fusion (21), which could enhance the quality of muscle leading to increased force production. Finally and importantly, future studies should also examine whether biological sex plays a potential mediating role as our study is limited by only examining young healthy males.

In conclusion, our findings from our secondary analyses show that exercise training lowers serum Li levels, but GY supplementation can rescue the losses. Interestingly, maintaining and/or elevating serum Li during exercise training is associated with greater gains in total strength and reductions in bone resorption. Since controlling for factors in GY such as protein and calcium weakened these associations, future studies should determine the specific effects of dietary Li alone on musculoskeletal structure and function.

## Data Availability Statement

The raw data supporting the conclusions of this article will be made available by the authors, without undue reservation.

## Ethics Statement

The studies involving human participants were reviewed and approved by Brock University Biosciences Research Ethics Board. The patients/participants provided their written informed consent to participate in this study.

## Author Contributions

RB and LS: data curation, formal analysis, methodology, investigation, validation, writing—original draft, and writing—review and editing. AJ and VF: conceptualization, methodology, investigation, data curation, project administration, resources, formal analysis, validation, and writing—review and editing. All authors contributed to the article and approved the submitted version.

## Funding

This work was funded through unrestricted research grants from Brock University to AJ and VF. RB was supported by a Match of Minds Scholarship from Brock University and LS was supported by a Canadian Institutes of Health Research Postdoctoral Fellowship (#164711). VF was supported by a Canada Research Chair (Tier II) in Tissue Plasticity and Remodeling Throughout the Lifespan. The publication of this study was supported by a Brock University Library Open Access Fund.

## Conflict of Interest

AJ reports grant funding from Dairy Farmers of Canada, Dairy Management Inc., non-financial support from Danone and Parmalat, and consultant and speaker fees from Dairy Farmers of Canada. LS reports grant funding from Dairy Farmers of Canada and salary support from Dairy Management Inc. The remaining authors declare that the research was conducted in the absence of any commercial or financial relationships that could be construed as a potential conflict of interest.

## Publisher's Note

All claims expressed in this article are solely those of the authors and do not necessarily represent those of their affiliated organizations, or those of the publisher, the editors and the reviewers. Any product that may be evaluated in this article, or claim that may be made by its manufacturer, is not guaranteed or endorsed by the publisher.
